# The butyrate-producing and spore-forming bacterial genus *Coprococcus* as a potential biomarker for neurological disorders

**DOI:** 10.1017/gmb.2023.14

**Published:** 2023-08-30

**Authors:** Fleur Notting, Walter Pirovano, Wilbert Sybesma, Remco Kort

**Affiliations:** 1 Amsterdam Institute for Life and Environment, Vrije Universiteit Amsterdam, Amsterdam, The Netherlands; 2 Center for Neurogenomics and Cognitive Research, Vrije Universiteit Amsterdam, Amsterdam, The Netherlands; 3 Microbiome Solutions GmbH, Münsingen, Switzerland; 4 ARTIS-Micropia, Amsterdam, The Netherlands

**Keywords:** *Coprococcus eutactus*, short-chain fatty acids, fibre fermentation, gut–brain axis, depression

## Abstract

The host–intestinal microbiome interaction has gained much scientific attention in the past two decades, boosted by advances in DNA sequencing and cultivation techniques. An accumulating amount of evidence shows that gut microbes play crucial roles in gut homeostasis, immune system education, and are associated with quality-of-life indicators. Beneficial health factors are associated with the digestion of dietary fibres in the colon and the subsequent production of short-chain fatty acids, including acetate, propionate, and butyrate. *Coprococcus* is a butyrate-producing genus in the phylum Firmicutes, and its abundance is inversely correlated with several neuropsychological and neurodegenerative disorders. Case–control studies provide strong evidence of decreased abundance of *Coprococcus* spp. in depressed individuals. The species *Coprococcus eutactus* has the unique capacity to use two separate pathways for butyrate synthesis and has been found to be depleted in children with delayed language development and adults with Parkinson’s disease. The combined literature on *Coprococcus* and the gut microbiota–brain axis points towards enhanced butyrate production and reduced colonisation of pathogenic clades as factors explaining its association with health effects. The genus *Coprococcus* is a promising candidate for a mental health biomarker and an interesting lead for novel dietary-based preventive therapies for specific neurological disorders.

## Graphical abstract

**Figure:**
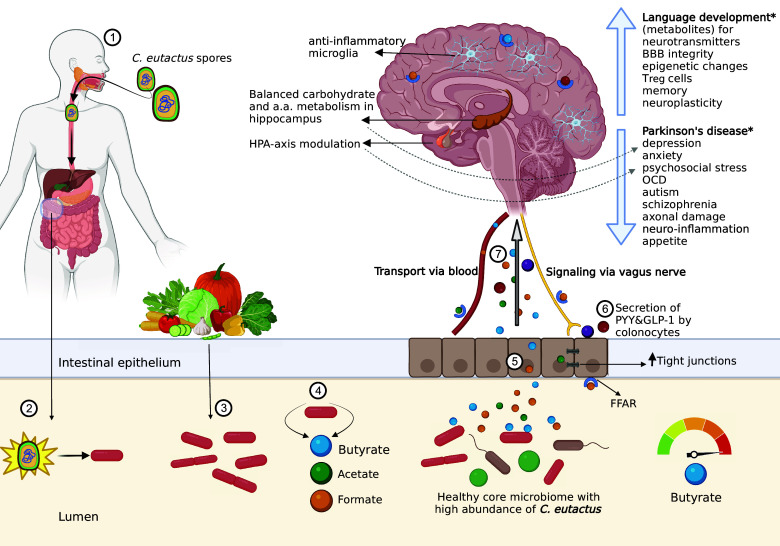
*Coprococcus* as a potential biomarker and modulator of neurological disorders. 1. Ingestion of *C. eutactus* spores during early infancy. 2. Germination of spores in the gut. 3. A fibre-rich diet strengthens colonisation of *C. eutactus.* 4. *C. eutactus* produces short-chain fatty acids (SCFAs) during fibre fermentation. It has multiple butyrate production pathways. 5. Colonocytes take up SCFAs via facilitated diffusion or via free fatty acid receptors (FFARs). Butyrate metabolism by colonocytes improves the epithelial barrier function. 6. The uptake of SCFAs leads to the secretion of glucagon-like peptide 1 (GLP-1) and anorexigenic peptide YY (PYY). These peptides suppress appetite and may have neurological effects. 7. The gut–brain axis constitutes SCFAs and peptides transported via the blood or SCFA signalling via the vagus nerve. The implied neurological effects are indicated in the top-right corner. *Specifically associated with *C. eutactus.* a.a., amino acids; BBB, blood–brain barrier; HPA, hypothalamic–pituitary–adrenal; OCD, obsessive–compulsive disorder. Created with BioRender.com.

## Introduction

Bacterial members of the microbiota in the intestinal tract are important factors in homeostasis. By occupying their niche, they limit the risk that pathogens, such as *Clostridium difficile* (Deshpande et al., 2013) or pathobionts (Kamada et al., 2013), can accumulate and invade the host. Additionally, they provide useful nutritional sources, such as vitamins and essential amino acids, and generate important metabolites from dietary fibres through their enzymatic activity in the gut (Bäckhed et al., 2005). Most bacteria in the gastrointestinal tract reside in the large intestine, with the remainder primarily found in the small intestine and stomach (Cummings and Macfarlane, 1991; Lawley and Walker, 2013). Bacteroidia (phylum Bacteroidetes, Gram-negative) and Clostridia (phylum Firmicutes, Gram-positive) are two dominant classes (The Human Microbiome Project Consortium, 2012). Together with Gammaproteobacteria, Actinomycetia, Synergistetia, Fusobacteriia, and Verrucomicrobiae, they almost complete the bacterial composition of the gastrointestinal tract (Browne et al., 2017). Most of these species are obligatory anaerobes.

To establish a mutualistic relationship between the gut microbiota and the human host and to maintain tolerated residency in the gut, extensive co-evolution with our immune system has preceded (Ley et al., 2008; Lee and Mazmanian, 2010). Continuous cross-talk between the microbiota genera and the innate and adaptive immune systems ensures tolerance (Saeed et al., 2019), and not only shapes our immune system locally but also systemically from young infancy (Mazmanian et al., 2005). Indeed, many studies have linked an individual core biome to health status. Dysbiosis has been associated with metabolic diseases, such as inflammatory bowel disease (IBD; DeGruttola et al., 2016), obesity (Greenblum et al., 2012), and type II diabetes mellitus (DM; Qin et al., 2012). The proposed mechanisms partly rely on abnormal levels of short-chain fatty acids (SCFAs) synthesised by bacterial phyla through fibre fermentation. SCFAs are taken up by colonic cells of the host as a high-energy source (Donohoe et al., 2011) and induce anti-inflammatory effects (Parada Venegas et al., 2019). An imbalance in SCFAs production may lead to chronic inflammation and subsequent progression to cancer (Chang and Parsonnet, 2010). In addition to cancer development (Sánchez-Alcoholado et al., 2020), the gut microbiota composition has been linked to metabolic diseases, allergic airway disease, autoimmune disorders, cardiovascular disease, and neurological disorders (Durack and Lynch, 2019), the latter in agreement with the notion of the gut–brain axis (Cryan et al., 2020).

The gut–brain axis encompasses direct interactions between the microbiota and central nervous system (e.g., via the vagus nerve) and indirect interactions, including immune-modulatory pathways and those mediated by microbial production of neurotransmitters and their precursors (Cryan et al., 2020). SCFAs play multiple roles in indirect interactions (O’Riordan et al., 2022). In this context, the anaerobic, Gram-positive and SCFA-producing bacterial species *C. eutactus* shows significant negative correlations with depression (Valles-Colomer et al., 2019), autism spectrum disorder (Andreo-Martínez et al., 2020), and Parkinson’s disease (Keshavarzian et al., 2015), among others. Additionally, *Coprococcus* spp. have been associated with beneficial health factors, such as circulating antioxidant indolepropionic acid (IPA; Menni et al., 2019) and omega-3 polyunsaturated fatty acids (Noriega et al., 2016), and are negatively correlated with ileal Crohn’s disease (Baumgart et al., 2007; Vacca et al., 2020). Therefore, it is a bacterial genus of interest because of its connection to homeostasis and the gut–brain axis. However, to date, there has been no systematic overview of the role of *Coprococcus* spp. in health and neurological disease states. This review summarises information on *Coprococcus* spp., with special attention paid to *C. eutactus*, in relation to the gut–brain axis, and evaluates the unique properties of *C. eutactus*, considering its potential as a biomarker organism for mental health. With growing insight into interactions between the host and the gut microbiome, the identification of “beneficial” and “harmful” gut bacteria at the onset of neuropsychological or neurodegenerative disorders may be possible. Moreover, this insight may aid in the development of preventive dietary interventions and treatment options for neuropsychiatric disorders.

## Properties of the genus *Coprococcus* and its member *Coprococcus eutactus*

### The genus *Coprococcus*

**Figure 1.**
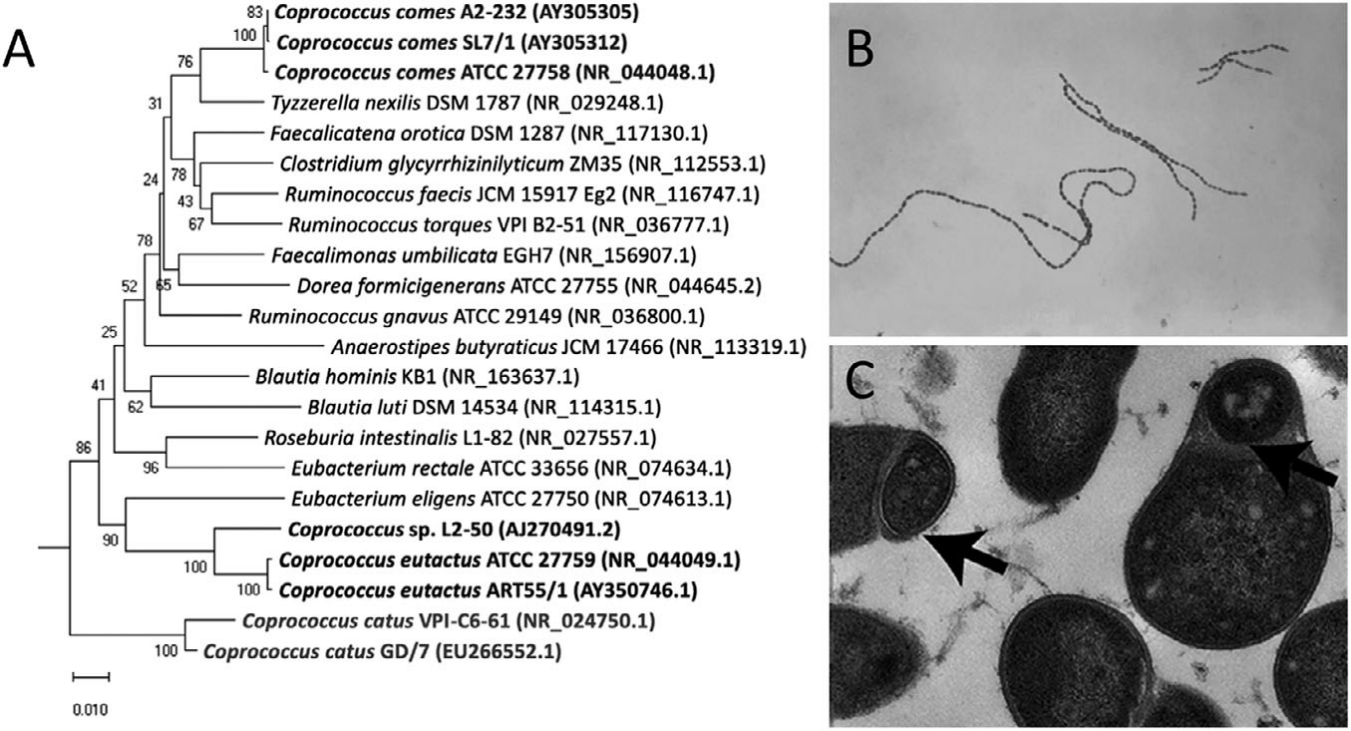
Phylogenetic tree and microscopic characteristics of *Coprococcus.* (A) Evolutionary relationships of *Coprococcus* species and their closest human gut bacteria relatives. Bootstrap values are shown at branches. Image retrieved from Alessi et al. (2020). (B) Gram-stained photomicrograph depicting a number of chains of Gram-positive, anaerobic, coccoid *Coprococcus eutactus* bacteria. Image retrieved from the Public Health Image Library (1976). (C) Transmission electron microscopy image of cultured *C. eutactus* cells. Arrows indicate spore ultrastructures. Image retrieved from Browne et al. (2016).

Members of the bacterial *genus Coprococcus* were first isolated and identified in human faeces (Holdeman and Moore, 1974). *C. eutactus*, *C. catus*, and *C. comes* are recognised as Gram-positive, obligate anaerobic cocci that produce butyric and acetic acids by fermentation of carbohydrates, and are classified in the phylum Firmicutes, class Clostridia, order Eubacteriales, and family *Peptococcaceae* (Holdeman and Moore, 1974). Bacterial taxonomy was previously based on similarities in morphology and functional features; however, the use of 16S ribosomal RNA (16S rRNA) gene sequencing techniques showed different clustering, resulting in the reclassification of the *Coprococcus* genus in the family *Lachnospiraceae* (Ezaki et al., 1994; Ludwig et al., 2009; Ezaki, 2015). Although grouped into a single family, *Coprococcus* spp. show large phylogenetic distances within their genus members (Vacca et al., 2020; Figure 1A). A potential new genus member, *Coprococcus phoceensis*, which clustered closely with *C. comes*, was recently isolated from the colon of a patient with Crohn’s disease (Bonnet et al., 2019). A 2021 study indicated five potential new *Coprococcus* species members based on 16S rRNA gene sequencing of faecal samples combined with symmetric pairwise average nucleotide identity values (ANI) and metabolic pathway network analyses (Nogal et al., 2021). Three of the proposed novel species clustered closely with *C. eutactus* and shared several metabolic features. However, these newly identified species have not yet been officially characterised. It should be noted that the frequently used approach to define bacterial species based on genotypic and phenotypic properties is controversial, as it leads to an underestimation of true bacterial diversity (Dykhuizen, 1998; Staley, 2006). In particular, taxonomic assignment using 16S rRNA gene sequencing is limited because genetically distinct bacteria can share similar or even identical 16S rRNA sequences (Jaspers and Overmann, 2004). The use of novel techniques with a higher resolution, such as multilocus sequence analysis and whole genome sequencing, can help gain a broader insight into the diversity of the *Coprococcus* genus and the speciation process.

### Cell morphology

Holdeman and Moore (1974) first described *Coprococcus* spp. as nonmotile, Gram-positive cocci, with *C. eutactus* ranging from 0.7 to 1.3 μm in diameter usually observed in pairs, although long strings of *C. eutactus* have since also been found (Figure 1B). *C. catus* showed a slightly elongated morphology, with an average cell size of 1.1 by 1.7 μm, presented in pairs forming long chains (Holdeman and Moore, 1974). *C. comes* was found to be an elongated coccus with tapered ends, with an average cell size of 1.6 by 2.3 μm, although the size range showed quite some variability. Cells occurred either singly, in duplets, or in chains of 4–20 elements. The cell wall peptidoglycan of *Coprococcus* spp. contains meso-diaminopimelic acid at position three with no interpeptides (Ezaki et al., 1994). Although coccoid morphology is the simplest bacterial form to generate, the ancestral bacterial morphology is that of a rod, not a sphere, and several lines of evidence suggest that environmental selective pressure is the driving force behind this shape change (Yang et al., 2016). Size minimisation and coccoid morphology *of Coprococcus* spp. could have improved their ability to colonise the mucosal surface of the gut and reduce their susceptibility to host defences.

### Genome

Type strains of *Coprococcus* were isolated from human faeces and registered in the American Type Culture Collection (ATCC) database. The type strain of *C. eutactus* (ATCC 27759) carries a genome of 3.11 Mb containing 2,654 genes and 1,380 hypothetical proteins (ATCC, 2020; Benton et al., 2021). Its G+C content is considered relatively low at 43.1% (Ezaki et al., 1994). The genome of *C. comes* (ATCC 27758) is somewhat larger with 3.38 Mb, 3,177 genes, and 1,616 hypothetical proteins (ATCC, 2021). The G+C content was determined to 42.52%. Finally, the genome of *C. catus* (ATCC 27761) contains 3.66 Mb, 3,346 genes and 1,726 hypothetical proteins, with a G+C content of 43.01% (ATCC, 2022). *Coprococcus* spp. are considered part of the core microbiome with carbohydrate-fermenting properties in the healthy human gut (King et al., 2019), and genes for SCFA synthesis pathways have been found in different *Coprococcus* species (see section “Production and synthesis pathways”). Additionally, glycoside hydrolase (GH) genes for cellulose breakdown (see section “Oxygen sensitivity and cultivation”) have been specifically identified in the genome of *C. eutactus* (Alessi et al., 2020). Sporulation genes are described in the following section.

### Life cycle and growth requirements

*Coprococcus* spp. reside in the human colon as obligate anaerobic bacteria and have been isolated from oral samples of 45% of healthy adult subjects (Segata et al., 2012). Once shed through faeces, intestinal bacteria temporarily encounter environmental stress before being transmitted to a new host. Rapid colonisation by direct human-to-human contact, modulation of metabolism, and high activity of repair pathways are strategies to counteract environmental challenges. The classes Clostridia and Bacilli within the Firmicutes phylum are known to produce spores and metabolically dormant structures to withstand atmospheric oxygen during environmental exposure (Browne et al., 2017). *C. eutactus* and its members have long been assumed to be non-sporulating bacteria (Collins et al., 1994); however, this has been disputed by Browne et al. (2016), who combined the culture-independent approach of metagenomic sequencing with bacterial culturing and estimated that 60% of the intestinal microbiota genera, representing approximately 30% of the total intestinal microbial abundance, produces spores. Selection of bacterial spore-forming species by treating faecal samples with ethanol resulted in the isolation of 66 ethanol-resistant species, including members of the genus *Coprococcus*; *Coprococcus* spp. contain 53% of the known sporulation genes, and transmission electron microscopy revealed typical spore structures in cultured *C. eutactus* (Browne et al., 2016; Figure 1C).

Analysis of the distribution of the main sporulation-associated gene families *cot*, *spo, sps*, and *ssp* within the genomes of *Lachnospiraceae* revealed that 27 out of 84 sporulation genes of the spore-forming bacterium *Bacillus subtilis* had no known homologues in *the sequenced genomes of Lachnospiraceae* (Meehan and Beiko, 2014). Interestingly, 29 of the remaining 57 genes were present in the majority of gut-associated *Lachnospiraceae*, including *C. eutactus* and *C. comes*, but were completely absent from the rumen and oral-associated family members of *Lachnospiraceae,* suggesting that the acquisition of sporulation genes is a specific ecological adaptation to the gut environment; representatives of all the main sporulation-associated gene families, including *cot* (2 genes), *spo* (31 genes), *sps* (5 genes), and *ssp* (4 genes), were identified in the genomes of *C. eutactus* and *C. comes* (Meehan and Beiko, 2014). Once excreted from the body in faeces, *Coprococcus* spp. should be able to tolerate the local environment and colonise a new host. Dormant bacterial spores are extremely resistant and can cope with the toxic effects of atmospheric oxygen, ultraviolet radiation, lack of nutrients, adverse temperatures, and desiccation (Browne et al., 2017). Bacterial spores have great application potential as probiotic food supplements, as they offer the benefit of higher survival rates during passage through the acidic stomach and better stability during processing and storage than the widely used lactic acid bacteria (Bader et al., 2012).

Common bile acids (taurocholate, glycocholate, and cholate*)* were evaluated for their stimulatory effects on spore germination in the gut. In particular, taurocholate proved to be a potent germinant for all sporeformers, increasing the culturability of spores from commensal bacteria by up to 70,000-fold. Bile acids did not affect the culturability of non-spore-forming bacteria. Browne et al. (2017) proposed a bile acid-triggered “germination cue” to initiate colonisation by intestinal spore-forming bacteria. Spore formation ensures environmental survival and human-to-human transmission, whereas germination ensures bacterial persistence in the human host.

*Coprococcus* spp. are obligatory anaerobic bacteria. In a systematic investigation of the effects of atmospheric oxygen exposure on human intestinal microorganisms, 200 anaerobic bacterial strains were isolated (Brusa et al., 1989). Of this subset, 30 strains showed high O_2_-intolerance, including *Coprococcus* species. Similar to other highly O_2_-sensitive strains, *C. comes* cells showed only 50% survival after 4–5 min of oxygen exposure and 100% mortality after 100–120 min. The growth of highly O_2_-intolerant species needs to occur under strict anoxic conditions, making the cultivation of *Coprococcus* spp. tedious. Moreover, *C. eutactus*, *C. catus*, and *C. comes* have different preferences for nutrients and conditions. Holdeman and Moore (1974) reported the growth of these three species under anoxic conditions in rumen fluid-glucose-cellobiose agar roll tubes. *C. eutactus* showed poor or no growth in peptone yeast (PY) broth without fermentable carbohydrates, while PY-glucose cultures had the most abundant growth. Growth resulted in the production of formic, butyric, lactic, and acetic acids, with abundant hydrogen release. When the medium was enriched with pyruvate, formate, and acetate were formed. Growth occurred equally well at 37 C and 45 C but was poor to moderate at 25 C and 30 C. The researchers noted in their paper that *C. eutactus* was easily recognizable by its production of formate, lactate, and butyrate from glucose, and its uniform fermentation of these substrates.

*Coprococcus catus* displayed a different growth behaviour. Substrate utilisation was restricted, and slight to moderate growth was observed in PY broth. In contrast to glucose, fructose led to abundant growth. This resulted in the production of large amounts of butyric and propionic acids with hydrogen formation. There was usually no growth at 30 C, whereas the best growth occurred between 37 C and 45 C. The third species*, C. comes*, showed moderate growth in PY broth without carbohydrates. PY-glucose cultures showed abundant growth, resulting in the production of lactate, butyrate, and acetate. Interestingly, the metabolic pathways used did not result in hydrogen release. Additionally, cultures grown on PY-pyruvate and *C. comes* could use a range of carbohydrates as substrates. The temperature for optimal growth was 37 C; most strains grew well at 45 C, but not at 30 C. According to Holdeman and Moore (1974), the fermentation of arabinose and xylose helps to differentiate it from the other two species in the genus.

The ability to use lactate for growth also differs among species. Reichardt et al. (2014) confirmed that both *C. eutactus* ATCC 27759 and *C. comes* ATCC 27758 could not grow on lactate, but *C. catus* GD/7 was able to grow with 25 mM lactate on YCFA medium containing 30 mM acetate. Lactate utilisation also occurred in the presence of 10 mM fructose, and propionate was the main product in all lactose-supplemented cultures. A different metabolic pathway was used during growth on fructose in the absence of lactate, resulting in butyrate production and the net consumption of acetate. In a recent study, Alessi et al. (2020) examined the ability of *Coprococcus* spp. to utilise carbohydrate substrates by measuring the optical density and acidification. GH is associated with cellulose breakdown, with two GH9-encoding and four GH5-encoding genes in *C. eutactus* ART55/1 and a related strain, *Coprococcus* sp. L2-50. However, both strains failed to utilise cellulose. In other *Coprococcus* species, no GH genes were present, apart from a single GH5 gene, in one of the examined *C. comes* strains. As reported by Holdeman and Moore (1974), *C. eutactus* displayed the highest promiscuousness for carbohydrate utilisation among *Coprococcus* members. *Coprococcus* L2-50 exhibited growth on glucose, cellobiose, β-glucan, and lichenan but limited growth on potato starch. *C. eutactus* ART55/1 and *C. eutactus* ATCC 27759 were also able to grow on glucomannan, galactomannan, galactan and potato starch, and weak fermentation of mannan was detected. *C. comes* and *C. catus* showed more restricted carbohydrate utilisation; *C. comes* strains only grew well on glucose, while *C. catus* GD/7 showed limited growth on potato starch. The known substrate utilisation of *Coprococcus* spp. is listed in Supplementary Table S1.

## *Coprococcus* species in the human intestinal environment

### The impact of Coprococcus on gut homeostasis

In the gut, 90% of resident microbes are obligate anaerobes, constituting the main SCFA producers, and their presence is associated with homeostasis. *Coprococcus* spp. produce butyrate (Vital et al., 2014) together with other fermenters from the bacterial class of anaerobic Clostridia. *C. eutactus* significantly contributes to the pool of intestinal butyrate, listed at the fourth position in butyrate producers in the microbiota community, regarding the dominant metabolic butyrate pathway, the acetyl-CoA pathway (Vital et al., 2014). *Faecalibacterium prausnitzii*, *Ruminococcaceae,* and *Eubacterium rectale* were positioned at numbers 1, 2, and 3, respectively, whereas *C. comes* only at position 10 and *C. catus* at position 18. However, the exact ordering of species needs to be treated with caution, since analyses were only based on stool samples from only 15 individuals.

Microbiota-produced butyrate is oxidised to carbon dioxide by mature colonocytes for energy (ATP) generation, which is necessary for the transport of sodium (Na^+^) enabling water absorption. Butyrate metabolism requires colonocytes to consume high levels of oxygen, creating a hypoxic epithelial surface (Rivera-Chávez et al., 2017). Disruption of this hypoxic surface has pathophysiological consequences: antibiotic treatment with streptomycin preferentially depletes the Clostridia class, thereby reducing butyrate production and increasing partial oxygen pressure, leading to diffusion of oxygen into the intestinal lumen. This creates a window of opportunity for oxygen-tolerant microbes and is associated with the uncontrolled expansion of facultative anaerobic bacteria such as *Enterobacteriaceae*, including pathogenic *Escherichia coli* or *Salmonella enterica* species (Rivera-Chávez et al., 2016; Rivera-Chávez et al., 2017). Oxidative stress is known to have pathogenic effects through the generation of reactive oxygen species (ROS) and reactive nitrogen species (RNS). Million and Raoult (2018) investigated the link between faecal redox potential and human gut microbiome. A significant positive correlation was found between faecal redox potential and the predominance of aerotolerant species. Healthy subjects showed a lower redox potential than diseased subjects due to different forms of malnutrition.

Homeostasis and redox balance in the intestine are achieved by cross-feeding mechanisms. To understand the interdependency of the different microbial clades in the gut and the role of *Coprococcus* spp., the fermentation process must be considered in more detail. Fermentation, similar to aerobic respiration, involves the breakdown of nutrients by redox reactions (the oxidation of one molecule coupled to the reduction of another) to generate ATP. Hydrogen (H_2_), carbon dioxide (CO_2_), and methane (CH_4_) are produced by colonic microbes that ferment dietary components while producing SCFAs (Carbonero et al., 2012). In aerobic respiration, oxygen serves as the terminal electron, and nutrients are fully oxidised. However, during fermentation, nutrients are incompletely oxidised and the reduced fermentation products function as terminal electron acceptors. To maintain the redox balance, the reduced pyridine (NADH) and flavin (FADH) nucleotides must be reoxidised. This reaction is the primary source of hydrogen gas in the colon. Hydrogen production by hydrogenogenic microbes ensures the efficiency of fermentation, but hydrogen accumulation inhibits further fermentation owing to thermodynamic restrictions. Therefore, the simultaneous oxidation of hydrogen by hydrogenotrophic (H_2_-utilising) microbes that generate energy through anaerobic respiration forms a crucial companionship for SCFA-producing colonic microbes, such as *Coprococcus* species. The hydrogen-consuming clades consist of reductive acetogens, methanogenic archaea, and sulphate-reducing bacteria. Notably, a disturbed hydrogen economy has been associated with IBD (King et al., 1998; Carbonero et al., 2012).

In summary, under homeostatic conditions, the colonic epithelium is hypoxic because of the metabolism of SCFAs by mature colonocytes. With the loss of butyrate-producing microbes, such as *Coprococcus* spp., oxygen diffuses into the lumen and supports the expansion of pathogenic oxygen-tolerant microbes. The production of SCFAs by obligate anaerobes results in the release of H_2_ into the gut. The co-occurrence of hydrogen-producing and hydrogen-utilising microbes is crucial for ensuring continued SCFA production. When the balance of this companionship is disturbed, butyrate production is diminished, and oxygen levels in the gut rise, leading to reduced epithelial barrier function and opportunity for colonisation of pathogenic clades.

### Acquirement, prevalence, and abundance of the Coprococcus genus in the gut

An individual’s core microbiome shapes itself from birth and displays characteristic patterns according to age and changes in diet (Yao et al., 2021; see Textbox). Colonisation by the *Coprococcus* genus in the early life stages has been demonstrated in at least 87% of 3–4 months’ old Canadian infants (*n* = 24), with a low abundance of approximately 0.8% (Azad et al., 2013). It should be noted that differential abundance analysis is controversial throughout microbiome research, neglecting total microbial load, or absolute number of microorganisms (Morton et al., 2019), and core species are not necessary the most abundant ones. Many highly significant synergistic interactions have been identified among low-abundance species forming a connected network (Claussen et al., 2017). However, the prevalence and abundance of *Coprococcus* spp. increased over time as both were positively correlated with age. For example, *Coprococcus* spp. could be detected in the faecal samples of 78.2% of Chinese children at the age of 3 (*n* = 729) with a mean abundance of 0.21% (Niu et al., 2020). In contrast, Kostick et al. (2015) demonstrated large fluctuations with a rise and drop below the baseline of *C. eutactus* in children up to 3 years from Finland and Estonia (*n* = 33). The children were selected based on type I DM susceptibility (HLA DR-DQ alleles conferring risk of T1DM), which could explain the different findings compared with the Chinese cohort. Geographical factors should also be considered, since the intestinal microbiota of infants from different countries exhibit different types of microbe-dominant compositions (Kuang et al., 2016). These consist of the P-, A-, and F-types, referring to abundances in Proteobacteria, Actinobacteria, and Firmicutes, respectively. Most Chinese infants are P-type, whereas children from Western countries are predominantly A-type, which could impact the initial colonisation of *Coprococcus* species. The P-type was also determined in a cohort of Ugandan children (*n* = 139), and similar to the observations in the Chinese cohort, the prevalence of *C. eutactus* increased with age; *C. eutactus* prevalence increased from 24 to 36 months from 62 to 81% (Kort et al., 2021).

*Shaping the gut microbiome from birth*

- Intestinal bacterial colonisation occurs through vertical transmission (e.g., faecal and vaginal microbiota via vaginal delivery and via breast milk), and through horizontal transmission (via food and faecal-oral route).
- *Lactobacillus* and *Bifidobacterium*, present in breast milk and the maternal faecal and vaginal microbiome, initially colonise the neonatal intestine (Milani et al., 2015; Yao et al., 2021).
- In the first months of life, the gut microbiome is dominated by facultative anaerobes. However, when the infant gut gradually becomes anaerobic, a shift to strict anaerobes is observed (Bokulich et al., 2016; Niu et al., 2020).
- First, closely resembling their mother’s microbiome (Milani et al., 2015), the gut microbiome diversity increases dramatically, until an adult-like equilibrium is reached within the first 3 years of life (Yatsunenko et al., 2012).
- During this time period, Bacteroidetes, Firmicutes, Proteobacteria, and Actinobacteria are the dominant phyla.
- When nutrition changes to solid foods, composition and diversity of the infant gut microbiota transition accordingly. While the relative abundance of Bacteroidetes increases over time, Proteobacteria and Actinobacteria significantly reduce in number from birth to the age of 2 (Niu et al., 2020; Yao et al., 2021).
- Intestinal residency may already start from the foetal stage, indicated by studies showing that the placenta harbours nonpathogenic commensal microbiota from the Firmicutes, Tenericutes, Proteobacteria, Bacteroidetes, and Fusobacteria phyla (Aagaard et al., 2014; Ding et al., 2017).

Odamaki et al. (2016) analysed faecal samples from 367 healthy Japanese individuals between the ages of 0 and 104 years and found a distinct, adult-associated co-abundance group (CAG) of bacterial species that were increased compared to the infant and elderly microbiome compositions. The adult-like CAG included *Lachnospiraceae*, *Blautia*, *Roseburia*, *Faecalibacterium*, *Lachnospira*, and *Coprococcus.* These findings have also been observed in other large cohort studies. *Coprococcus* spp. were identified as part of the 20 adult core genera in the Belgian Flemish Gut Flora (FGFP) cohort (*n* = 1106), and they were listed in the 14 core genera (prevalence of 95% or higher) from the combined Western and non-Western cohorts (*n* ≈ 4,300; Falony et al., 2016). *Coprococcus* was present in 99.73% of the samples in the FGFP cohort, whereas the coverage for the non-Western cohort was lower (84.09%). King et al. (2019) constituted a healthy human reference microbiome list (based on faecal and metagenomic faecal samples of approximately 100 adults) with 157 organisms. Scoring both a prevalence of 100%, *C. eutactus* ART55/1 and *C. catus* were included in this list of the healthy core microbiome, with a mean relative abundance of 0.68 and 0.37%, respectively. *Coprococcus* spp. were also included in the top 33 most abundant taxa in the FGFP cohort, ranking ninth place when scaled by the rarefied abundance data (Falony et al., 2016). However, the abundance of *Coprococcus* differs largely per person, as *Coprococcus* spp. were listed in the top 10 contributors of interindividual variation in microbiota composition, resting on changes in the relative abundance of the core taxa. Shetty et al. (2017) specifically identified *C. eutactus* as a member of the core microbiota in adult subjects across mainland Europe, UK/Ireland, and the USA (*n* = 401), with a prevalence of 100%. Moreover, *C. eutactus* was ranked fifth in relative abundance, while the other *Coprococcus* members were absent from the list of 80 core bacterial species.

Later in life, new shifts in microbiota configuration occur (Odamaki et al., 2016), where seven genera, including *Coprococcus*, are negatively correlated with age and are found to be significantly lower in centenarians than in elderly people aged 80–99 years (Fang et al., 2015). In summary, *Coprococcus* spp. are acquired by age and can occur in the early months of life. The mechanism of colonisation is unclear, but may be facilitated through spore-mediated horizontal transmission. *C. eutactus* is part of the healthy Western adult core microbiome (Shetty et al., 2017), but with advanced age, the abundance of the *genus Coprococcus* decreases again (Fang et al., 2015).

### Association between host genetics and Coprococcus abundance in the gut microbiome

A number of studies have shown evidence for associations between the abundance of *Coprococcus* in the gut microbiome and host genotypes, particularly with respect to IBD pathogenesis. A genetic association study performed by Rausch et al. (2011) in Caucasian populations, including healthy subjects and patients with Crohn’s disease (*n* = 47), indicated an association between the human *FUT2* gene located on chromosome 19 and the abundance of *Coprococcus*, *Alistipes,* and unclassified *Lachnospiraceae* in the human gut (Rausch et al., 2011). More specifically, through a host genotyping and 16S V1–V2 rRNA taxonomic profiling approach, the authors detected associations between the three OTUs and individuals with Crohn’s disease (CD) that were homozygous for the loss-of-function allele “A” at position rs601338. An association between the abundance of *Coprococcus* and genetic variation in a risk locus linked to CD was also observed Li et al. (2016), who performed a genotyping analysis of large cohorts (*n* = 16,249) of IBD patients and healthy subjects. They found that a well-known pleotropic missense variant in the *SLC39A8* gene (rs13107325) was correlated with the abundance of five genera in the microbiome (*Anaerostipes*, *Coprococcus*, *Roseburia*, *Lachnospira*, and *SMB53*) in individuals with CD and healthy controls. Missense variants have also been strongly associated with an increased risk of schizophrenia (Schizophrenia Working Group of the Psychiatric Genomics Consortium, 2014).

Although the aforementioned studies did not directly investigate the influence of host genetics on the microbiome in mental health-related diseases, there is increasing evidence linking the gut–brain axis to the development of both gastrointestinal and neurological diseases (Günther et al., 2021). The rationale behind this connection is that the metabolites produced by gut microbes and molecules released by the mucosal immune system in response to inflammation or infection (such as cytokines) both affect the integrity of the blood–brain barrier (BBB) and epithelial barrier, and thus influence CNS functions. This suggests that indirect mechanisms may exist through which gut bacteria modulate neurological diseases. Significant associations have been found between IBD and neurodegenerative and neuroinflammatory diseases. For example, a recent study by Villumsen et al. (2019) on a large Danish cohort *n* = 7,548,259), showed that IBD patients had a 22% increased risk of developing Parkinson’s disease (PD). Kosmidou et al. (2017) performed a large meta-analysis (total *n* = 1,086,430) indicating that both IBD and MS patients have more than 50% increased risk to have concomitant MS and IBD, respectively. In conclusion, recent studies have shown that indirect mechanisms through which gut bacteria modulate neurological diseases may exist. However, future research is needed to elucidate the interaction between the abundance of specific *Coprococcus* species, such as *C. eutactus*, and the host genome and how this impacts the direct or indirect development of mental disorders.

### Coprococcus species and general health associations

In a recent analysis of a large microbiome population cohort (FGFP, *n* = 1,054), both *Faecalibacterium* and *Coprococcus* bacteria were consistently associated with higher quality of life (QoL) indicators, addressed by mental and physical scoring indicators (Valles-Colomer et al., 2019). Indeed, *Coprococcus* spp. have been implicated as beneficial health factors in previous studies; microbiome alpha-diversity was found to be positively correlated with circulating acetate levels in a cohort of 948 women from TwinsUK, while four bacterial genera, including *Coprococcus*, were linked to higher serum acetate levels (Nogal et al., 2021). The latter study also identified a negative correlation between visceral fat and serum acetate level. Similarly, in 1,018 middle-aged women from the TwinsUK cohort, microbiome alpha diversity was positively correlated with serum IPA levels, a metabolite produced by intestinal microbes from dietary tryptophan (Menni et al., 2019). IPA regulates the gastrointestinal barrier function (Venkatesh et al., 2014) and is associated with a reduced risk of type II DM (de Mello et al., 2017). Along *with F. prausnitzii*, Mollicutes (phylum Tenericutes), *Ruminococcaceae* (mostly butyrate-producing bacteria), *and C. eutactus* and its members were positively correlated with IPA serum levels (Menni et al., 2019).

Preeclampsia (PE) is a pregnancy-specific hypertensive disorder that impairs maternal and foetal health, but it also shares similarities in clinical features and pathogenic mechanisms with metabolic diseases such as DM (Valdés et al., 2014; Liu et al., 2017). In studies investigating faecal samples from South-Chinese (*n* = 100) and Australian (*n* = 213) pregnant women, *Coprococcus* spp. were negatively correlated with the PE status (Liu et al., 2017; Altemani et al., 2021). However, in a Chinese study, *C. catus* was specifically decreased, whereas an association was found for the pooled *Coprococcus* genus in Australian women. The authors of the first study suggested the ability of *C. catus* to produce propionic acid with its inhibiting effect on cholesterol synthesis as a mode of mechanism, while the Australian group demonstrated additional evidence for butyrate-related mechanisms; *Coprococcus* abundance significantly and positively correlated with the abundance of genes encoding bacterial butyrate formation. In addition, circulating butyrate levels were lower in PE women prior to the development of symptoms.

A Finnish study investigated the intestinal microbiota composition of 6-month-old infants in relation to eczema and found that the severity of symptoms correlated inversely with microbiota diversity and the abundance of butyrate producers (Nylund et al., 2015). Early infants received a 3-month dietary intervention with hydrolysed casein formula with or without *Lactobacillus rhamnosus* GG supplementation. Clinical symptoms (assessed by SCORAD values) were alleviated in all infants, which coincided with increased microbiota diversity. Moreover, decreased SCORAD values correlated with an increased abundance of eight butyrate-producing bacterial species, with the highest effect size observed for the increased abundance of *C. eutactus.* Similarly, in a small cohort of children (1–6 years old) with atopic dermatitis, alterations were found in the gut microbiota compared to controls, characterised by lower microbiota diversity and increased abundances of *Faecalibacterium*, *Oscillospira*, *Bacteroides*, *Parabacteroides*, and *Sutterella* (Reddel et al., 2019). In line with a previous study, a reduced abundance of SCFA-producing bacteria was observed, including *Coprococcus.* Lastly, the *Coprococcus* genus has been negatively associated with ileal CD, whereas the ileitis mucosa was enriched in sequences for the facultative anaerobic bacterium *E. coli* (Baumgart et al., 2007).

## Short-chain fatty acids

### Production and synthesis pathways

The beneficial health effects of the gut microbiota are mostly attributed to the production of SCFAs. SCFAs are found in high concentrations in dairy products and can thus be directly taken up through food or indirectly as the fermentation products of our intestinal microbiota (O’Riordan et al., 2022). All *Coprococcus* species produce SCFAs acetate and butyrate, but differ in the metabolic pathways and substrates used, as well as the amounts and ratios of the produced SCFAs (Holdeman and Moore, 1974). Only *C. catus* is characterised by its large production of propionate (Holdeman and Moore, 1974; Reichardt et al., 2014).

**Figure 2.**
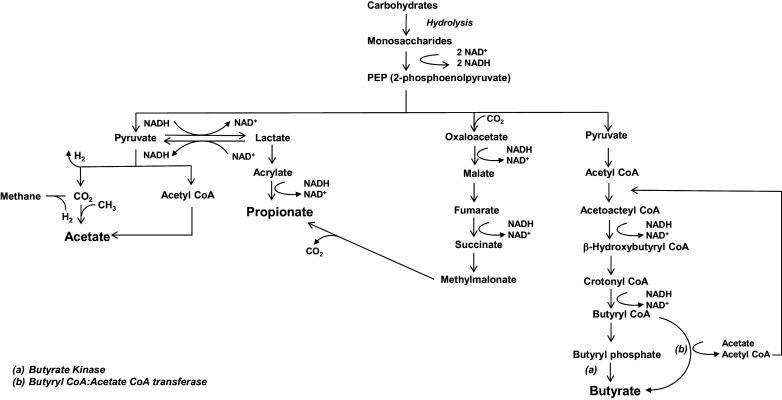
Degradation pathways for carbohydrates into short-chain fatty acids (SCFAs). Overview of carbohydrate break-down into SCFAs. Image reproduced from Parada Venegas et al. (2019).

As the main products of anaerobic fermentation of indigestible polysaccharides by microbiota, SCFAs are small carboxylic acids containing an aliphatic tail with a maximum chain length of six carbon atoms (Parada Venegas et al., 2019). Acetate (C2), propionate (C3), and butyrate (C4) are the most abundant SCFAs found in the large intestine (McNeil et al., 1978; Cummings et al., 1987; Rios-Covian et al., 2020), and the substrates for bacterial SCFA production include resistant starch (i.e., starch and products of starch degradation not absorbed in the small intestine), oat and wheat bran, cellulose, and pectin (Parada Venegas et al., 2019). Figure 2 provides an overview of the major routes for SCFA formation. Notably, resistant starch forms an important source for butyrate synthesis (Champ, 2004). Members of Bacteroidetes are responsible for the production of bulk acetate and propionate, whereas Firmicutes are the main butyrate producers in the human gut (Louis and Flint, 2017).

**Figure 3.**
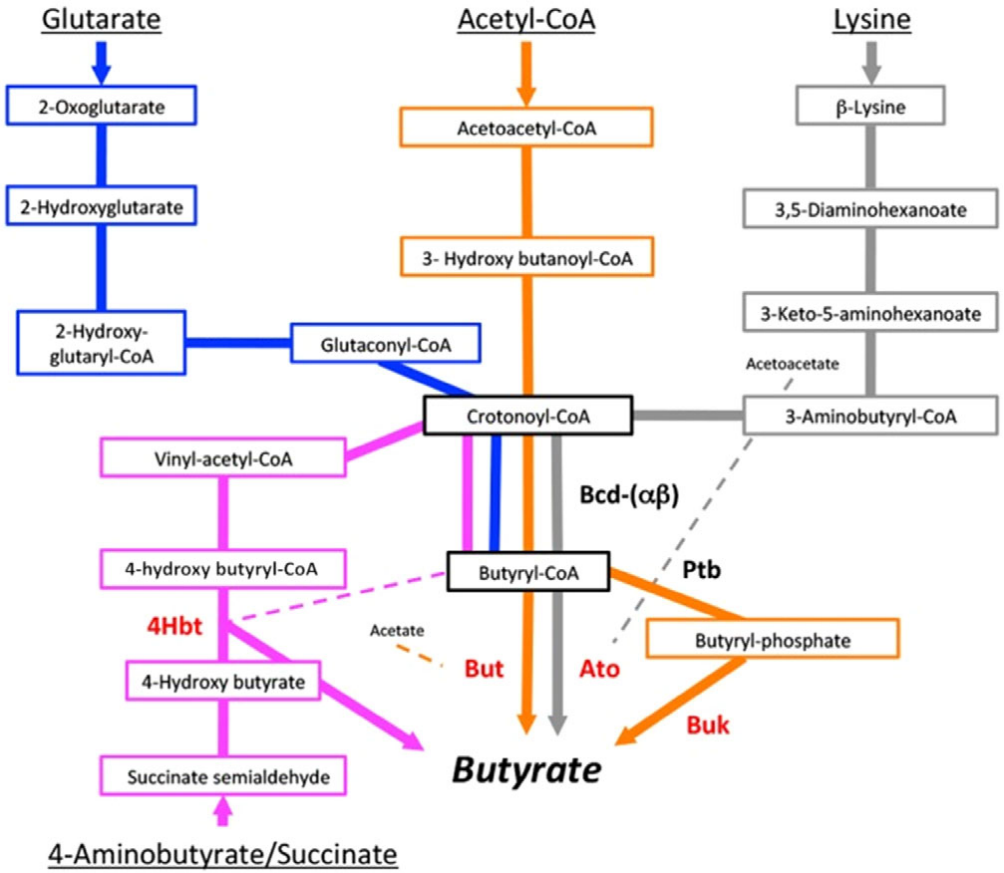
The butyrate synthesis pathways. The four bacterial butyrate synthesis pathways were retrieved from a study by Vital et al. (2014). Major enzymes are indicated and terminal genes are highlighted in red. Bcd, butyryl-CoA dehydrogenase (including electron transfer protein α and β subunits); Ptb, phosphate butyryltransferase; 4Hbt, butyryl-CoA:4-hydroxy-butyrate CoA transferase; But, butyryl-CoA:acetate CoA transferase; Ato, butyryl-CoA:acetoacetate CoA transferase; Buk, butyrate kinase.

The microbial synthesis pathways of SCFAs were first described by Miller and Wolin (1996), and additional pathways have since been discovered. Butyrate formation predominantly occurs from carbohydrate hydrolysis and subsequent glycolysis; however, butyrate can also be synthesised from organic acids and amino acids (Louis and Flint, 2017). Butyrate production occurs via either the acetyl-CoA, glutarate, 4-aminobutyrate, or lysine pathways (Vital et al., 2014; Figure 3). The acetyl-CoA pathway accounts for the bulk of bacterial butyrate formation and related genes are found in the majority of butyrate producers (Vital et al., 2014). Genes for the lysine pathway are also present in many phyla, whereas the 4-aminobutyrate- and glutarate-based pathways are the least abundant. Several isolates have been reported to carry genes for multiple butyrate pathways, indicating that butyrate synthesis plays a prominent role in energy conservation.

These pathways merge at a central step, where crotonyl-CoA is transformed to butyryl-CoA, thereby creating a proton motive force to conserve energy in ATP for the bacterium. Thus, butyrate production not only restores the redox balance by regeneration of NAD^+^ from NADH + H^+^ produced upstream in the carbohydrate breakdown for ATP conversion, but also results in additional ATP production (Louis and Flint, 2009; Vital et al., 2014). The final conversion to butyrate is performed by various butyryl-CoA transferases such as butyryl-CoA:acetate CoA transferase (But). The latter requires external acetate as a co-factor, donated through cross-feeding mechanisms (Duncan et al., 2002, 2004). Alternatively, butyryl-CoA is phosphorylated and transformed into butyrate via butyrate kinase (Buk), resulting in additional ATP synthesis (Miller and Wolin, 1996; Vital et al., 2014; Louis and Flint, 2017).

As mentioned earlier, the majority of intestinal butyrate producers belong to the Firmicutes phylum, particularly Clostridia clusters IV and XIVa, the latter of which includes the *Coprococcus genus* (Rivière et al., 2016). These clusters contain Gram-positive, highly oxygen-sensitive, and strictly anaerobic saccharolytic bacteria. Considering their relatively high abundance in the human colon, *Faecalibacterium prausnitzii* (*Clostridium* cluster IV) and *Eubacterium rectale* (*Clostridium* cluster XIVa) are expected to contribute more significantly to intestinal butyrate production than *Coprococcus* spp. However, specific synthesis pathways and substrates place *Coprococcus* spp. in a unique position. All *Coprococcu*s spp. are butyrate producers, with *C. catus* containing only the But and *C. comes* only the Buk genes; *Coprococcus eutactus* contains both the But and Buk genes (Vital et al., 2014), allowing butyrate production under less restricted conditions and substrate availability, and potentially higher energy conservation by utilisation of the butyrate-kinase pathway. Importantly, only a few other human gut-associated taxa (*Clostridium* sp. L2–50, *Ruminococcaceae* bacterium D16, and *Acidaminococcus* sp. D21) are known to encode both But and Buk genes (Vital et al., 2014). In addition, Vital et al. (2014) listed *C. eutactus* as the fourth bacterial contributor to the dominant butyrate pathway, the acetyl-CoA pathway, highlighting its role in colonic butyrate formation.

Three distinct pathways for propionate production have been described: succinate, acrylate, and propanediol pathways, using polysaccharides, organic acids, lactate, and amino acids as substrates (Reichardt et al., 2014). Since the succinate pathway is present in the abundant phylum Bacteroidete*s*, as well as in certain Negativicutes bacteria, it is assumed to be the most dominant route for propionate production (Reichardt et al., 2014). In the succinate pathway, the decarboxylation of methylmalonyl-CoA to propionyl-CoA is coupled with sodium transport across the membrane, driving ATP generation via sodium-translocating ATPase. In the acrylate pathway, the lactoyl-CoA dehydratase gene serves as a marker, which was identified in *C. catus* (Reichardt et al., 2014). Through isotype-labelling experiments, *C. catus* was found to convert virtually all lactate to propionate via the acrylate pathway. Interestingly, colonic bacteria seem to encode genes for either butyrate synthesis or propionate synthesis, whereas only two bacterial species are known to produce both butyrate and propionate as end products of carbohydrate fermentation; *C. catus* produces butyrate when grown on fructose and shifts to propionate production when grown on lactate. Similarly, *Roseburia* *inulinivorans* generates butyrate from glucose, but converts fucose into propionate. Among the other *Coprococcus* members, *C. comes* and *C. eutactus* lack propionate synthesis genes, are not able to grow on lactate, and instead produce butyrate from sugar fermentation.

As acetate is the most produced SCFA in the gut, anaerobic fermentation of carbohydrates often entails the breakdown of monosaccharides into acetyl coenzyme A (acetyl-CoA) and subsequent acetate production, generating ATP by substrate-level phosphorylation (Zhang et al., 2021). Five pathways have been reported, all for which acetyl-CoA serves as a precursor (Figure 2), although acetyl phosphate may alternatively function as a high-energy substrate in one pathway. In their investigation of microbiota-produced acetate, Nogal et al. (2021) assigned three acetate pathways to both *C. eutactus* and *C. comes*, and two to *C. catus*, but the authors did not refer to specific metabolic pathways. Therefore, genomic data analysis is required to resolve the pathways for acetate biosynthesis by *Coprococcus* species.

## *Coprococcus* in neurological and mental disease

### Coprococcus associations with neurophysiological states and mental disorders

In addition to general health-associated factors, studies have linked the *Coprococcus* genus to multiple neurological and psychological disorders (Table 1; search methodology specified in the Supplementary File S2), where *C. eutactus* is specifically associated with Parkinson’s disease (PD; Petrov et al., 2017) and language development in children (Kort et al., 2021). The association between reduced relative abundance of *Coprococcus* spp. in the gut and PD was found 2 years earlier in a smaller American cohort (Keshavarzian et al., 2015). Both studies indicated a reduced abundance of butyrate-producing bacteria *Blautia*, *Coprococcus*, and *Roseburia* in patients with PD, whereas putative pro-inflammatory bacteria, such as *Oscillospira*, were increased. Moreover, the American team showed a negative correlation between PD duration and the abundance of *Lachnospiraceae*, a bacterial family comprising many anti-inflammatory butyrate-producers, including *Coprococcus.* Additionally, they performed predictive metagenomics of the faecal microbiome community functionality and found that genes for lipopolysaccharide biosynthesis and type III bacterial secretion systems were higher in PD patients. These results support the theory that intestinal dysbiosis could trigger leakage of the intestinal barrier, subsequent inflammatory response, and oxidative stress, leading to alpha-synuclein (α-Syn) aggregation. Indeed, α-Syn deposits in the colon of patients with PD have been demonstrated already 2–5 years before the onset of PD symptoms. The mechanism of consequent neuroinflammation and α-Syn deposition in the brain is still highly speculative, but may involve direct pathways via the mesenteric nerve system, reduced anti-inflammatory responses in the CNS due to a shortage of microbiota-produced SCFAs, or even a prion-like spreading of the α-Syn aggregates to the brain.

**Table 1.** Summary of studies with significant correlation of *Coprococcus* genus and neurological disorders.

Genus species	Study focus	Finding	Cohort	Sample size (*n*)	*P*-value	Statistical test	Study
*Coprococcus*	Autism Spectrum disorder (ASD)	Lower relative abundance in autistic children	American children (age 3–16); 20 with ASD plus GI complaints, and 20 neurotypical GI symptom-free controls	40	0.001	Mann–Whitney *U* test	Kang et al. (2013)
*Coprococcus*	ASD	Higher relative abundance in autistic children	Children from Ecuador (age 5 to 12 years); 25 with ASD and 35 controls	60	< 0.05	Mann–Whitney *U* test followed by gneiss analysis	Zurita et al. (2020)
*Coprococcus*	Parkinson’s disease (PD)	Lower relative abundance in PD patients	American adults (age 46–82); 34 PD patients and 21 healthy controls	65	0.03	Kruskal–Wallis’ test	Keshavarzian et al. (2015)
*Coprococcus eutactus*	PD	Lower relative abundance in PD patients	Russian adults (Siberia, age 61–69); 89 PD patients, and 66 patients without parkinsonism (controls)	155	0.03	White’s *t-*test	Petrov et al. (2017)
*Coprococcus*	Schizophrenia	Lower relative abundance in schizophrenic individuals	Chinese (Han nationality) adults (age 18–65); 64 schizophrenia patients and 53 healthy controls	117	<0.05	Welch’s *t*-test	Shen et al. (2018)
*Coprococcus*	Epilepsy	Higher abundance in patients with drug-resistant than with drug-sensitive epilepsy	West-Chinese individuals (age 5–50); 42 drug-resistant and 49 drug-sensitive epileptic patients, 65 healthy controls	156	<0.05	LEfSe > 2	Peng et al. (2018)
*Coprococcus*	Depression	Lower relative abundance in patient groups compared to controls	Chinese adult cohort, Peking region; 40 IBS-D patients (mean age 38.5), 15 depression patients (mean age 44.8), 25 comorbidity patients (mean age 39.0), and 20 healthy controls (mean age 43.9)	100	<0.001	Wilcoxon rank-sum test	Liu et al. (2016)
*Coprococcus*	Depression	Depleted in depressed individuals	Flemish adults (FGFP cohort); mean age: 50.9 years	1,054	0.0015	Covariance test	Valles-Colomer et al. (2019)
*Coprococcus*	Depression	Depleted in depressed individuals	Dutch adults (LLD cohort); mean age: 57.9 years	1,063	0.013	Covariance test	Valles-Colomer et al. (2019)
*Coprococcus catus*	Depression	Lower relative abundance in patient groups compared to controls	Chinese adults (age 18–65); 40 depressed individuals and 22 healthy controls	62	0.000331	Statistical test validated by false discovery rate > 0.1	Li et al. (2022)
*Coprococcus*	Depression	Negative association with depressive symptoms	Dutch adults (RS cohort); age range 45–87 years	1,054	0.000909	Linear regression model	Radjabzadeh et al. (2022)
*Coprococcus*	Depression	Negative association with depressive symptoms	Dutch adults (HELIUS cohort); age range 19–71 years	1,539	0.0012	Linear regression model	Radjabzadeh et al. (2022)
*Coprococcus*	Major depressive disorder (MDD)	Lower relative abundance in MDD patients	Han Chinese adults (Beijing region); 27 MDD patients (mean age 48.7) and 27 healthy controls (mean age 42.3)	54	<0.001	LEfSe > 2 validated by Kruskal–Wallis test	Huang et al. (2018)
*Coprococcus*	MDD	Lower relative abundance in MDD patients	Chinese female cohort (age range 18–65 years); 62 patients and 46 healthy controls	62	0.0000246	LEfSe > 2 validated by Mann–Whitney *U* test	Chen et al. (2021)
*Coprococcus*	Bipolar depression (BD)	Lower relative abundance in BD patients	Chinese cohort (age range 14–61 years); 52 BD patients and 45 HCs	97	0.0046	LEfSe > 2 validated by ANOVA	Hu et al. (2019)
*Coprococcus*	Mood improvement in obese individuals	High baseline *Coprococcus* abundance predicts response to inulin intervention and mood improvement	Belgian obese adults (age range 18–65 years); 43 patients on 3-month prebiotic treatment, 43 patients in placebo group	86	< 0.05	Mixed models analysis (HSD Tukey test)	Leyrolle et al. (2021)
*Coprococcus*	Generalised anxiety disorder (GAD)	Lower relative abundance in GAD patients	Han Chinese adult cohort (age range: 18–65 years); 36 active GAD patients and 24 healthy controls	60	<0.05	LEfSe > 2	Chen et al. (2019)
*Coprococcus*	GAD	Negative correlation of baseline *Coprococcus* abundance with reduction in SAS score after 1-month pharmaceutical treatment	Han Chinese adult cohort (age range: 18–65 years); 36 active GAD patients and 24 healthy controls	60	<0.05	LEfSe > 2	Chen et al. (2019)
*Coprococcus*	Obsessive–compulsive disorder (OCD)	Lower relative abundance in OCD patients	Spanish adults (age range 18–71); 38 OCD patients and 33 healthy controls	71	<0.05	LEFSe >2	Domènech et al. (2022)
*C. eutactus*	Language development	Predicts at 24 months language development at 36 months	Rural Ugandan children; sampled at 20–24 months and 36 months	139	<0.001	All subsets regression	Kort et al., 2021
*Coprococcus*	Neuromyelitis optica spectrum disorders (NMOSDs)	Lower relative abundance in NMOSD patients	West-Chinese adults; 20 NMOSD patients (mean age of 48.15) and 20 healthy controls (mean age of 47.65)	40	<0.05	Mann–Whitney *U* test	Shi et al. (2020)

Abbreviations: LEfSe, linear discriminant analysis effect size; SAS, self-rating anxiety scale.

SCFA synthesis and hydrogen formation are inherently linked (see section “The impact of *Coprococcus* on gut homeostasis” ), and intestinal microbiota from PD patients have been demonstrated to produce 2.2-fold less hydrogen compared to controls (Suzuki et al., 2018). Despite a promising pilot study (Yoritaka et al., 2013), the administration of exogenous hydrogen (by hydrogen water) to patients with PD has been proven unsuccessful in a larger-scale investigation (Yoritaka et al., 2018). Suzuki et al. (2018) claimed that the imbalance in the hydrogen economy in patients with PD may be dependent on many classes of SCFA producers and cross-feeders, and does not only depend on butyrate production. As the authors mentioned, a large proportion of hydrogen was also produced by the reduction of formate. Microbiome metabolism in the gut accounts for approximately 50% of the formate pool in human serum (Pietzke et al., 2020). Formate is involved in many physiological processes including purine synthesis, sterol and tryptophan metabolism, and methylation. Interestingly, circulating urate levels are reduced in PD patients (Ascherio et al., 2009). Notably, formate acts as a precursor for purine synthesis, and urate as a product of purine catabolism (Pietzke et al., 2020). Indeed, in early onset PD, mitochondrial formate production is impaired and serum urate levels increase. Although further studies are warranted, we speculate that SCFAs other than butyrate play important roles in the aetiology of PD. Apart from butyrate, *C. eutactus* produces large amounts of formate, possibly contributing to the association between *Coprococcus* spp. and PD pathology.

Eight studies demonstrated lower relative abundance of *Coprococcus* spp. in individuals suffering from depression or depression-like symptoms (Liu et al., 2016; Huang et al., 2018; Hu et al., 2019; Valles-Colomer et al., 2019; Chen et al., 2021; Leyrolle et al., 2021; Li et al., 2022; Radjabzadeh et al., 2022), establishing a link between the *Coprococcus* genus and the gut–brain axis. The large Flemish and Dutch cohorts showed a significant negative correlation between *Coprococcus* spp. and depression when findings were corrected for antidepressant medication (Valles-Colomer et al., 2019), which was recently replicated in two other large Dutch cohorts (Radjabzadeh et al., 2022). However, in the latter study, *Ruminococcaceae* UCG005 was the main predictor of depressive symptoms, whereas the *Coprococcus* genus was not listed as an important predictor (Radjabzadeh et al., 2022). Interestingly, using Mendelian randomisation, the group identified a causal link between *Eggerthella* and depression. Another interesting finding has been reported by Liu et al. (2016) found a reduced abundance of *Coprococcus* spp. in patient groups, but also found high similarities between the intestinal microbiome composition of patients with inflammatory bowel syndrome (IBS) and depressed individuals. Together with the high observed comorbidity rate of IBS and depression, these results may indicate a common dysbiosis-related pathogenesis; intestinal inflammation could disturb the communication between the enteric nervous system and the CNS. Similarly, obesity and depression often coincide, and Leyrolle et al. (2021) stratified a cohort of obese adults in high and low intestinal abundance of *Coprococcus.* High-baseline *Coprococcus* predicted better mood improvement after a 3-month prebiotic inulin intervention compared to the placebo group and low-baseline group. However, the relative contribution of *Coprococcus* is difficult to estimate since the positive response was also correlated with an increased inflammatory profile, insulin resistance, and adiposity at baseline. Reininghaus et al. (2020) performed a 4-week multistrain probiotic plus biotin intervention in an inpatient setting for depressed individuals. *Ruminococcus gauvreauii*, *Coprococcus*, and β-diversity increased in the probiotic group after 28 days. Moreover, KEGG analysis showed elevated anti-inflammatory and metabolic pathways, including vitamin B1 and B6/7 metabolism. Beneficial effects on clinical parameters were observed, but were similar in the biotin and placebo groups. Four weeks may be insufficient to observe any differences in clinical outcome measures, but the study shows that appropriate probiotic treatment can elevate *Coprococcus* levels in the short term and simultaneously upregulate inflammation-regulating responses.

These studies share the finding that butyrate-producing bacteria, mostly from the Firmicutes phylum, are reduced in individuals with depression. Although all three *Coprococcus* spp. produce butyrate, *C. eutactus* may add more to the microbiota-produced butyrate pool owing to its availability of multiple butyrate-synthesis pathways and higher general abundance, and consequently confer a higher protective effect on the development of depression. However, a study in Polish females investigated the link between faecal SCFA levels and depression and found that acetate and propionate were negatively correlated with clinical parameters of depression (Skonieczna-Żydecka et al., 2018). Differences in absorption rates of individual SCFAs by colonocytes cannot be excluded, although they may explain inconsistencies in findings between faecal microbiome and faecal SCFA analysis. Other possible mechanisms, besides butyrate production, underlying the microbiome association with reduced susceptibility to depression, were proposed by Valles-Colomer et al. (2019). The authors analysed the association between microbiome features and the diagnosis of depression and QoL using two large population cohorts (Table 1). First, the authors assessed the neuroactive potential of the gut microbiota. More specifically, through a thorough literature search, they defined 56 gut–brain modules (GBMs), corresponding to distinct microbial pathways by which a neuroactive compound (a chemical that interacts with the human nervous system) is either synthesised or degraded. From a subsequent search in 532 microbial reference genomes of the human gastrointestinal tract, 15 GBMs could be detected within one or more *Coprococcus* spp. genomes included in the dataset (*C. eutactus* ATCC 27759, *C. comes* ATC 27758, *C. catus* GD/7, and *C.* sp. ART55/1). A summary of these results is presented in Table 2. Out of the 13 GBMs found in the *C. eutactus* strain, two GMBs for tryptophan synthesis and acetate degradation were not detected in the *C. comes* and *C. catus* strains. Instead, one GMB for isovaleric acid synthesis II was shown to be present in both the *C. comes* and *C. catus* strains, but not in the *C. eutactus* strain. Notably, the three GBMs for p-cresol synthesis, 17-beta-estradiol degradation, and isovaleric acid synthesis II were found in at least one of the available *Coprococcus* spp. genomes, although their corresponding functions have not yet been experimentally observed and described in the literature. In the microbiome-wide association study, Valles-Colomer et al. (2019) found significant covariation of 3,4-dihydroxyphenylacetic acid (DOPAC) synthesis potential with mental QoL scores in both the Flemish and Dutch cohorts. DOPAC is the metabolic end-product of dopamine conversion, and reduced DOPAC levels in cerebrospinal fluid have been observed in patients with PD. Interestingly, DOPAC synthesis potential was strongly associated with the relative abundance of *Coprococcus.* Although the *Coprococcus* spp. do not seem to synthesise DOPAC, one of the metabolic steps in the DOPAC synthesis pathway, the second step in the GBM, which converts 3,4-dihydroxyphenylacetaldehyde to DOPAC, is encoded in the genomes of *C. comes* and *C. catus.*

**Table 2.** Summary of the detected gut–brain modules (GBMs) in the genomes of *Coprococcus* spp. retrieved from Valles-Colomer et al. (2019).

Gut–brain module (GBM)	*C. eutactus* ATCC 27759	*C. comes* ATCC 27758	*C. catus* GD/7	*C*. sp. ART55/1
Tryptophan synthesis	○			○
Glutamate synthesis I	○	○	○	○
Glutamate synthesis II	○	○	○	○
p-Cresol synthesis	○	○	○	○
ClpB (ATP-dep chap protein)	○	○	○	○
17-beta-Estradiol degradation	○	○		○
Quinolinic acid synthesis	○	○	○	○
Quinolinic acid degradation	○	○	○	○
Isovaleric acid synthesis II		○	○	
*S*-Adenosylmethionine synthesis	○	○	○	○
Acetate synthesis I	○	○	○	○
Acetate degradation	○			○
Butyrate synthesis I	○	○		
Butyrate synthesis II			○	
Propionate synthesis II	○		○	○
	13	11	11	12

*Note*: For the GBMs indicated in grey, the corresponding functions have not yet been experimentally confirmed in the literature. The finding of only one butyrate-synthesis GBM for *Coprococcus eutactus* is in contrast with the findings from the study by Vital et al. (2014) showing that the same strain (ATCC 27759) contains two butyrate synthesis pathways, mediated by the But and Buk genes.

Kang et al. (2013) published findings on the reduced intestinal abundance of *Coprococcus* spp. and other fermenters in autistic children, paving the way for new insights into the pathogenesis of neurological disorders. This is in contrast with a recent study showing an increased abundance of *Coprococcus* spp. in autistic children compared to controls in a cohort of autistic children from Ecuador (Zurita et al., 2020). However, the effect size seemed low and Supplementary Material was not included in the analysis. A meta-analysis conducted by Iglesias-Vázquez et al. (2020) on gut microbiome composition included several small studies and indicated a cumulative, significant reduction in *Coprococcus* spp. in autistic children. Similarly, single studies on generalised anxiety disorder (GAD; Chen et al., 2019), schizophrenia (Shen et al., 2018), and obsessive–compulsory disorder (OCD; Domènech et al., 2022) have shown a reduced relative abundance of *Coprococcus* spp. in faecal samples of patients. The authors observed a general trend of increased taxa associated with oxidative stress and the reduction of SCFA-producing bacteria. In the OCD study, the authors also considered a DOPAC-mediated mechanism to explain the reduced abundance of *Coprococcus* spp. in patients, as dopaminergic transmission may be involved in the neurobiology of OCD (Domènech et al., 2022).

Peng et al. (2018) found alterations in gut microbiome composition between drug-resistant epileptic patients, drug-sensitive patients, and healthy controls. There was an increased relative abundance of *Coprococcus* spp. in drug-resistant patients, but while other studies confirmed increased Firmicutes relative to Bacteroides in refractory epileptic individuals, the associated taxa differ per study and findings on increased *Coprococcus* spp. abundance have not been replicated (Lum et al., 2020). *Coprococcus* and cognitive capacities have been linked in a recent study on language development in young Ugandan children. The presence of *C. eutactus* in faeces at 24 months could predict language development at 36 months (Kort et al., 2021). Comparing their findings to the PD microbiome-related studies, the authors hint at increased butyrate-production as an explanatory mechanism. However, additional studies demonstrating a reduced abundance of *Coprococcus* spp. and other neurodegenerative/neurocognitive disorders, such as Alzheimer’s disease, are lacking. The only exception may be a study on mindful awareness training in elderly patients diagnosed with mild cognitive impairment; cognitive impairment improved, and *Coprococcus* spp. were positively correlated with Colour Trails Test 2, Digit Span Backward and Block Design tests (Khine et al., 2020). Unfortunately, possible explanations for the mechanisms of such bidirectional brain–gut interactions have not been provided.

Neuromyelitis optica spectrum disorders (NMOSDs) are categorised as a group of autoimmune demyelinating diseases of the CNS that lead to visual loss and severe motor impairment. Shi et al. (2020) discovered significant differences in microbiota composition between patients and controls, and could distinguish patients by increased abundance of the pathogenic genera *Flavonifractor* and *Streptococcus*, and decreased abundance of commensal bacteria, including *Coprococcus* specie*s.* KEGG analysis showed that the observed gut microbiome alterations were related to the downregulation of pathways for photosynthesis, and thiamine (vitamin B1) metabolism. The authors hypothesised that thiamine deficiency may exert negative effects on Th1 and Th17 cell subpopulations and reduce the persistence of commensals by interfering with vitamin B1 metabolism, thereby contributing to the loss of intestinal barrier function. Additionally, lower levels of photosynthetic activity could partly explain the observation that patients with NMOSD display low levels of circulating vitamin D. Vitamin D deficiency may increase intestinal permeability even further, thereby enhancing the release of endotoxins in the circulatory system to promote the development of neuroinflammation.

In conclusion, a lower relative abundance of *Coprococcus* spp. is associated with mental and neurophysiological disorders, although a higher relative abundance has also been demonstrated in patients with autism and refractory epilepsy. *Coprococcus*-associated disorders cover a broad range of neurological syndromes or states, as recognised in a meta-analysis of gut microbiota composition and psychiatric disorders (Nikolova et al., 2021). Here, the authors could find “a transdiagnostic pattern of microbiota signatures” where “depleted levels of *Faecalibacterium* and *Coprococcus* and enriched levels of *Eggerthella* were consistently shared” between the analysed studies on psychiatric disorders. The combined findings suggest that the disorders are characterised by a reduction in anti-inflammatory butyrate-producing bacteria and an observed enrichment of pro-inflammatory genera. In this review, we analysed *Coprococcus* in association with cognitive and mental disorders, while excluding psychiatric disorders directly involving diet and nutritional habits (e.g., anorexia nervosa and bulimia). In addition, this review aimed to determine whether *C. eutactus* is specifically associated with certain mental health disorders. Only two studies specifically indicated an association of *C. eutactus* with PD or language development, whereas the other studies either did not specify the *Coprococcus* genus members or found associations with all three *Coprococcus* species. Without a clear distinction between the *Coprococcus* species in these studies, such an analysis is difficult to perform. This is further complicated because members of the *genus Coprococcus* may be poorly characterised in general (Nogal et al., 2021), and future studies may first endeavour to unravel the different phylogenetic branches within the genus and possibly categorise the genus into more individual species.

### Short-chain fatty acids and the gut–brain axis

As indicated in the previous section, the derived associations between the *Coprococcus* genus and neuropsychological disorders point towards SCFA-mediated mechanisms. To understand the various ways SCFAs may interact with the gut–brain axis, knowledge of receptors on host cells for SCFA uptake and signalling is crucial. Colonocytes use passive diffusion for SCFA uptake and facilitate SCFA diffusion to co-absorb SCFAs with Na^+^ or K^+^ cations (O’Riordan et al., 2022). The most important transporters are sodium-coupled monocarboxylate transporter (SMCT1) and proton-coupled monocarboxylate transporter (MCT) 1 and 4. MCT1 functions as the primary transporter for butyrate uptake in colonocytes and is upregulated by butyrate and fermentable carbohydrates (Parada Venegas et al., 2019). In humans, the proximal colon and ileum contain low expression of SCFA transporters, but MCT1 and MCT4 increase along the colon, reaching their highest number in the distal colon.

Even before SCFAs enter the systemic circulation, where they may exert effects on the brain, SCFAs can activate anti-inflammatory signalling cascades. SCFAs serve as ligands for various G-protein coupled receptors (GPCRs) and hydroxycarboxylic acid receptor 2 (GPR109a/HCAR2) in colonocytes (Parada Venegas et al., 2019; O’Riordan et al., 2022). The best characterised GPCRs are GPR43 and GPR41, commonly referred to as free fatty acid receptor 2 (FFAR2) and FFAR3, respectively. FFAR2 has a high affinity for acetate and propionate, whereas FFAR3 prefers longer fatty acid structures such as butyrate. FFAR2 and FFAR3 are not specifically expressed in intestinal epithelial cells, but are found in tissues such as the vasculature and kidneys, as well as in various immune cells (e.g., neutrophils, monocytes, and lymphocytes). Direct activation of the vagus nerve and enteric nervous system by SCFAs may be possible, since FFAR3 is expressed in the periportal afferent neural system, as well as in the enteric neural plexus and autonomic and sensory ganglia.

The proposed roles of SCFAs in brain physiology were described by O’Riordan et al. (2022). In summary, SCFAs may regulate microglial gene expression, maturation, morphology, and abundance. Acetate may alter (metabolites for) the neurotransmitters glutamate, glutamine, and γ-aminobutyric acid (GABA) as well as anorexigenic neuropeptide expression in the hypothalamus. Microbiota-derived butyrate may improve the integrity of the BBB by inducing higher levels of the tight junction protein occludin. Butyrate may also induce epigenetic changes by increasing histone acetylation and histone crotonylation in the brain. Additionally, butyrate epigenetically regulates microglial responses through the downregulation of pro-inflammatory mediators, while upregulating the expression of anti-inflammatory mediators. Propionate has been associated with reduced experimental autoimmune encephalomyelitis (EAE) and axonal damage through increased *T*_reg_ differentiation. The combination of propionate and butyrate regulates tryptophan hydroxylase expression and modulates intracellular potassium levels in the CNS.

Endothelial cells abundantly express MCT, which is the likely mode of transport for SCFAs to cross the BBB (Silva et al., 2020). FFAR3 has also been found in brain endothelial cells, suggesting a signalling-mediated pathway in which butyrate is involved in BBB protection (O’Riordan et al., 2022). Upon binding of SCFAs to FFAR2 and FFAR3, enteroendocrine colonocytes begin to secrete the anorexigenic hormone peptide YY (PYY) and glucagon-like peptide-1 (GLP-1), which are then transported to the brain via vagal afferents or circulating blood (O’Riordan et al., 2022). PYY and GLP-1 can influence appetite and food intake in the CNS. Notably, acetate increases hypothalamic neuropeptide expression, thereby reducing appetite. In a PYY and GLP-1 independent fashion, propionate has also been associated with reduced appetite and lower energy intake. PYY and GLP-1 have been shown to mediate cognitive processes in the reward centres of the brain, have anti-anxiety and anti-depressant properties, and enhance memory and neuroplasticity. However, the contributions of intestinal PYY and GLP-1 to these processes remain unclear. Furthermore, SCFAs may induce changes in the hormones insulin and leptin, and indirectly reduce ghrelin, an appetite-driving hormone. In addition to their metabolic influence, these hormones have been demonstrated to influence brain function, suggesting an extended repertoire of SCFA-mediated roles in cognitive processes.

In conclusion, the SCFAs butyrate, acetate, and propionate fulfil their roles in neurological function. These roles are mediated through the metabolism of neurotransmitters, epigenetic changes, and BBB integrity, as well as anti-inflammatory effects on microglia, microglial development, and influence on reward centres in the brain. More research is needed to clarify whether the supposed effects are derived from intestinal-derived SCFAs and whether these are directly taken up from the circulation or mediate indirect effects via the vagus nerve or peripheral immune system. In addition, connections between the neurological roles of SCFAs and neurophysiological or neuropsychological clinical outcomes should be further investigated.

## Dietary interventions

With a plethora of studies underlining the importance of microbiome composition to health, the possibility of altering the microbiome through nutritional interventions has been explored. After only 4 weeks of vegan diet intervention in healthy volunteers, *Coprococcus* levels were higher than those in participants on a meat-rich diet, for which relative abundance had decreased during that time period (Kohnert et al., 2021). Moreover, branched-chain amino acids (VAL, ILE, and LEU) were negatively associated with *Coprococcus* and *Dorea* in the vegan diet group. It is important to note that for most of the participants, microbiota composition remained similar during the 4-week trial, and the observed changes were attributed to a subgroup of participants. Thus, intra-individual differences between gut microbiota stability and diet change should be considered in nutritional diet studies. During a small randomised crossover nutritional trial, Tap et al. (2015) also observed differences in gut microbiota stability between healthy individuals. Diet change did not induce global effects among the participants; however, changing from a basal diet to a fibre-rich diet had a significant impact on the microbiota composition of each individual. High microbiome diversity was associated with higher microbiota stability, whereas increased fibre uptake modulated microbial metabolic pathways, including glycan metabolism. Moreover, microbial richness was positively correlated with *Coprococcus* spp. and *Prevotella* and with higher faecal concentrations of caproate and valerate.

Another study estimated the dietary habits of a general adult population and found that higher polysaccharide intake (characteristic of a Mediterranean diet) was correlated with a higher abundance of *Coprococcus* and *Bifidobacterium* (Garcia-Mantrana et al., 2018). In addition, higher total SCFAs were associated with greater consumption of plant-based nutrients, and adherence to the Mediterranean diet was positively correlated with total SCFAs. A 6-week intervention in healthy adults showed that daily supplementation with omega-3 resulted in a higher abundance of *Coprococcus* and *Bacteroides* spp., while the relative abundance of the fatty liver-associated *Collinsella* was reduced (Vijay et al., 2021). Moreover, butyrate production was increased, and *Coprococcus* spp. were positively associated with isobutyric acid and negatively associated with triglyceride-rich lipoproteins (VLDL and VLDL-TG). A randomised, double-blind, placebo-controlled crossover trial investigated the effect of a 4-week supplementation with the probiotic *Lactobacillus paracasei* DG. Probiotic intake resulted in an increased abundance of Proteobacteria and the *Coprococcus* genus, whereas *Blautia* decreased (Ferrario et al., 2014). Probiotic intervention modified enzymes involved in the metabolism of nicotinate and nicotinamide and the biosynthesis of folate (vitamin B9). The genus *Coprococcus* served as a predictor of folate biosynthesis genes along with the families *Prevotellaceae* and *Rikenellaceae* (phylum Bacteroidetes), and the genus *Phascolarctobacterium* (phylum Firmicutes).

In addition to certain dietary fibres and omega-3 lipids, vitamins, when directly delivered to the colon, have been shown to stimulate the growth of *Coprococcus* spp. In an *in vitro* model of the human gut microbiome, the relative abundance of *Coprococcus* spp. increased upon direct administration of vitamin A and vitamin D to the simulated microbiome environment; this increase was comparable to that observed with the prebiotic FOS (Pham et al., 2022). The results of the *in vitro* experiments were confirmed in a pilot human clinical trial. During a 4 weeks intervention period, administration of different types of vitamins in a colon-targeting delivery format showed an increase in the relative abundance of *Coprococcus* spp. compared to baseline (Pham et al., 2021). More specifically, vitamin D3 (60 μg cholecalciferol/day) resulted in an increase in *C. comes* (*p* = 0.042) compared with baseline. Similarly, the combination of vitamin B2 (75 mg riboflavin/day) and C (500 mg ascorbic acid/day) increased the relative abundance of *Coprococcus* spp. (*p* = 0.02). Supplementation with vitamin A (250 μg retinol equivalents/day) showed a trend of increased *Coprococcus* spp. levels, in line with the *in vitro* results (Pham et al., 2021). The concentration of SCFAs, including butyrate, increased depending on the type and dose of the administered vitamins.

The direct delivery of vitamins with antioxidative properties in the colon not only provides vitamins to the gut microbiome community but also reduces the gut redox potential, which diminishes oxidative stress and facilitates a more favourable environment for strictly anaerobic microorganisms (Million and Raoult, 2018). Taken together, the abundance of *Coprococcus* spp. seems to be relatively easily regulated by the type of food intake or at least seems to reflect the actual nutritional status. However, high inter-individual differences in microbiome changes owing to dietary interventions should be considered. The presumed beneficial health effects and increased abundance of *Coprococcus* spp. are often linked to increased SCFA production.

## Discussion

In this review, the *Coprococcus* genus, including the species *C. eutactus*, *C. comes*, and *C. catus*, has been described with attention to its morphologic and metabolic features, as well as its connection to human health and mental state. Numerous studies have shown positive correlations between *Coprococcus* abundance and increased QoL indicators. In particular, the link between the depletion of *Coprococcus* and depression has been well established and verified by large cohort studies. Although *Coprococcus* has consistently demonstrated a low abundance in intestinal, neuropsychological, and neurodegenerative disorders, almost all studies have shown multi-bacterial associations, stressing the need to obtain better insight into microbial communities and how they evolve and interact. In general, a reduction in anti-inflammatory butyrate-producing bacteria, such as *Faecalibacterium* and *Coprococcus*, and enrichment of pro-inflammatory genera were observed. This review focuses on the genus member *C. eutactus*, as it has multiple pathways for butyrate synthesis and is assumed to significantly contribute to the intestinal butyrate pool. However, only specific correlations between *C. eutactus* and PD and between *C. eutactus* and language development were found, whereas most studies reported associations between the *Coprococcus* genus. The latter can be attributed to either the lack of species resolution in the profiling techniques used, or the beneficial contribution of all *Coprococcus* spp. members. This may be explained by the fact that all *Coprococcus* members produce butyrate or via independent mechanisms, such as the potential of *C. catus* and *C. comes* to synthesise DOPAC, a metabolite implicated in PD. Besides butyrate, other SCFAs, such as propionate and formate, may play crucial roles in gut–brain interactions and could be attributed to *Coprococcus* associations with mental health conditions. In particular, the role of formate is poorly understood and requires further investigation.

With a plethora of studies underlining the beneficial health effects of SCFAs, numerous studies have attempted to increase intestinal SCFA-producing bacteria through dietary interventions. *Coprococcus* abundance can be rapidly increased by fibre, galacto-oligosaccharides, or omega-3 fats supplementation, as well as by probiotic administration, paving the way for *Coprococcus*-mediated nutritional interventions to prevent or alleviate intestinal or neuropsychological disorders. However, results on the clinical outcomes of such dietary interventions are still conflicting, and baseline microbiome composition may affect the individual response of microbiota compositional change. Moreover, whether these nutritional changes also result in long-lasting changes in gut microbiome composition has not been firmly established.

## General conclusion

The *Coprococcus* genus shows promise as a biomarker of gut–brain health, but future research should be aimed at characterising the host environment and microbial communities in which *Coprococcus* spp. thrive and function and evaluating the effects of *Coprococcus*-targeted interventions.

## Supporting information

Notting et al. supplementary material 1 Notting et al. supplementary material

Notting et al. supplementary material 2 Notting et al. supplementary material

## Supplementary material

The supplementary material for this article can be found at http://doi.org/10.1017/gmb.2023.14.

## Research transparency and reproducibility

All data and methods in this review are included in the journal article and in the Supplementary Files S1 and S2. The images, degradation pathways, and synthesis pathways in Figures 1–3, respectively, have been displayed in their original form and have been retrieved and reproduced from sources referenced in the figure legends; all third party content is free of copyright or has been published under a Creative Commons Licence.
