# Supplementary material for: The butyrate-producing and spore-forming bacterial genus Coprococcus as a potential biomarker for neurological disorders
Source: Gut Microbiome (Camb). 2023 Aug 30;4:e16. doi: 10.1017/gmb.2023.14 (PMC11406416; doi:10.1017/gmb.2023.14)
Supplement: Notting et al. supplementary material 1 — Notting et al. supplementary material [file S2632289723000142sup001.docx]

**Supplementary material**

**This is supplementary material for the review by Fleur Notting, Walter Pirovano, Wilbert Sybesma and Remco Kort (2023) he butyrate-producing and sporeforming bacterial genus Coprococcus as a potential biomarker for neurological disorders.**

**Table S1. Substrate utilization of the *Coprococcus* genus**

^1^ Information from Holdeman & Moore^35^

^2^ Information from Alessi et al.^46^

Y; growth observed, N; no growth, V; variable growth detected amongst isolates, w; weak growth. When two letters assigned then observations were different between the isolates. Between brackets []; divergent observation by Alessi et al.
