# Supplementary material for: The butyrate-producing and spore-forming bacterial genus Coprococcus as a potential biomarker for neurological disorders
Source: Gut Microbiome (Camb). 2023 Aug 30;4:e16. doi: 10.1017/gmb.2023.14 (PMC11406416; doi:10.1017/gmb.2023.14)
Supplement: Notting et al. supplementary material 2 — Notting et al. supplementary material [file S2632289723000142sup002.docx]

**Supplementary information on search methodology**

# This is supplementary material for the review by Fleur Notting, Walter Pirovano, Wilbert Sybesma and Remco Kort (2023) The butyrate-producing and sporeforming bacterial genus *Coprococcus* as a potential biomarker for neurological disorders.

Relevant articles for Table 2 “Summary of studies with significant correlation of *Coprococcus* genus and the gut-brain axis” were searched on PubMed through the following key words during September 19 – December 22, 2022:

(microbiome) AND (depression)

(microbiome) AND (gut-brain)

((Coprococcus) AND (fecal)) AND (depression)

((gut) OR (gastrointestinal) OR (intestinal) OR (feacal) OR (fecal) OR (stool)) AND ((microbiome) OR (microbiota) OR (bacteria) OR (dysbiosis))

AND (depression)

AND (depress*)

AND (mdd)

AND (bipolar)

AND (mania)

AND (anxiety)

AND (psychosis)

AND (schizophrenia)

AND (obsessive compulsive disorder)

AND (ocd)

AND (ptsd)

AND (post-traumatic stress disorder)

AND (adhd)

AND (attention deficit hyperactivity disorder)

AND (autism)

AND (ASD)

AND (Parkinson*)

AND (PD)

AND (epilepsy)

AND (cognition)

AND (dementia)

AND(Alzheime*)

AND (neurodegeneration)

AND (language)

AND (neuroinflammation)

AND (mood)

**Filters applied:**

Human

English

2003-2022

Case Reports

Clinical Study

Clinical Trial

Comparative Study

Meta-Analysis

Observational Study

Randomized Controlled Trial

**Inclusion criteria:**

(test) cohort n>20

Only significant P-values (<0.05) for association with relative abundance of *Coprococcus*

**Exclusion criteria:**

Psychological disorders directly linked to eating habits or disorders

Associations linked to self-reported symptom(s) of psychological disorder (e.g. “anxiety feeling” in depression)
